# Oligomerization and tyrosine nitration enhance the allergenic potential of the birch and grass pollen allergens Bet v 1 and Phl p 5

**DOI:** 10.3389/falgy.2023.1303943

**Published:** 2023-12-05

**Authors:** Janine Fröhlich-Nowoisky, Nadine Bothen, Anna T. Backes, Michael G. Weller, Ulrich Pöschl

**Affiliations:** ^1^ Multiphase Chemistry Department, Max Planck Institute for Chemistry, Mainz, Germany; ^2^ Division 1.5 - Protein Analysis, Federal Institute for Materials Research and Testing (BAM), Berlin, Germany

**Keywords:** allergy, Bet v 1, Phl p 5, nitration, dimers, oligomers, peroxynitrite, air pollution

## Abstract

Protein modifications such as oligomerization and tyrosine nitration alter the immune response to allergens and may contribute to the increasing prevalence of allergic diseases. In this mini-review, we summarize and discuss relevant findings for the major birch and grass pollen allergens Bet v 1 and Phl p 5 modified with tetranitromethane (laboratory studies), peroxynitrite (physiological processes), and ozone and nitrogen dioxide (environmental conditions). We focus on tyrosine nitration and the formation of protein dimers and higher oligomers via dityrosine cross-linking and the immunological effects studied.

## 1. Introduction

Tyrosine nitration and dityrosine cross-linking are post-translational protein modifications that occur under oxidative conditions. Tyrosine nitration involves the replacement of a hydrogen atom on the aromatic ring of the amino acid with a nitro group, while dityrosine cross-linking refers to the formation of covalent bonds between two tyrosine residues resulting in protein dimers and oligomers. These modifications affect protein structure and function and are associated with various diseases (1–3). They serve as biomarkers of oxidative damage in proteins, providing insight into the role of oxidative stress in disease development and progression (4–6). These tyrosine modifications also play a crucial role in altering the immune responses to allergens and may contribute to the increasing prevalence of allergic diseases and their association with traffic-related air pollution (7–12).

There are a variety of airborne allergen sources including plant pollen, fungal spores, and other biological aerosol particles. The allergenic proteins are released into the atmosphere when these particles rupture in response to humidity, exposure to anthropogenic air pollutants such as ozone (O${}_{3}$) and nitrogen dioxide (NO${}_{2}$), or mechanical influences (13–20). In the atmosphere, the proteins can react with reactive oxygen and nitrogen species (ROS/RNS), peroxyacetyl nitrate, or undergo photo-oxidation by UV radiation (10, 21–27). These reactions promote chemical protein modifications such as tyrosine nitration and dityrosine cross-linking via the formation of radical intermediates. In particular, summer smog conditions with high O${}_{3}$ and NO${}_{2}$ concentrations have been shown to efficiently nitrate and cross-link proteins within hours to days (21, 28–30). Air pollutants such as O${}_{3}$ and NO${}_{2}$, and particulate matter can also induce or enhance oxidative stress and inflammatory processes leading to the formation of endogenous ROS/RNS such as peroxynitrite (ONOO${}^{-}$), which is the main mediator of physiological tyrosine nitration and oligomerization (10, 31–34). In addition to the naturally occurring tyrosine nitration and cross-linking reactions, artificial protein nitration and dityrosine formation can be achieved by reaction with tetranitromethane (TNM), a standard laboratory reagent. While the TNM nitration lacks biological relevance, its usage in laboratory studies provides valuable insights into the consequences of tyrosine nitration and cross-linking in a protein molecule (8, 9, 28, 29, 35, 36). In addition to tyrosine nitration and dityrosine cross-linking, the reaction of proteins with oxidants can also result in other tyrosine modifications such as tyrosine nitrosylation, in the modification of other amino acids, and in protein degradation (29, 37–40).

The birch pollen allergen Bet v 1 and grass pollen allergen Phl p 5 are major airborne allergens in Central Europe (41–45). Both allergens serve as important diagnostic markers for genuine birch or grass pollen sensitization (44–47). In recent years, several studies have investigated the reactions of nitrating agents with the two allergens. Here, we summarize relevant findings on the chemical modification of these allergens by TNM, peroxynitrite, polluted air, O${}_{3}$/NO${}_{2}$ gas mixtures, and their effects on innate and adaptive immune responses and discuss future research perspectives.

## 2. Tyrosine nitration

The proportion of nitrated tyrosine residues in a protein can be represented by the tyrosine nitration degree (ND) and provides information about the extent of nitrosative and oxidative damage in a protein. It is defined as the ratio of nitrated tyrosine residues to the total number of tyrosine residues of a protein molecule. The ND of a protein can be determined by a variety of chromatographic, immunochemical, and mass spectrometry-based methods (28, 48–53).

For Bet v 1, the reaction with TNM yielded nitration degrees of up to 70% compared to peroxynitrite with up to 50% and to O${}_{3}$/NO${}_{2}$ achieving up to 22% nitration (29). The reaction of Bet v 1 with polluted air resulted in lower nitration degrees of ∼10% but shows that the allergen can be nitrated under environmental conditions (21). In contrast to Bet v 1, the grass pollen allergen Phl p 5 has received less attention with respect to tyrosine nitration. Only recently, Backes et al. (30) reported Phl p 5 nitration degrees of up to 40 % for the reaction with ONOO${}^{-}$ and up to 10% for O${}_{3}$/NO${}_{2}$ exposure.

The determination of the total nitration degree is particularly useful for kinetic investigations of tyrosine nitration, but not all tyrosine residues of a protein are nitrated with the same efficiency (52). Factors that determine the selectivity of tyrosine for the nitration reaction are the protein structure and the position of the tyrosine residues, the nitrating agent and the reaction conditions (10, 52, 54). Exposure of the aromatic ring to the surface of the protein, the location of the tyrosine within a loop structure, and its proximity to an adjacent negative charge favor nitration (55).

Determining the preferred nitration sites is particularly important for understanding the impact of nitration on protein functionality and interactions with the immune system. The total ND can be misleading when specific, biologically relevant tyrosine residues are highly nitrated while others remain unmodified as a single highly nitrated tyrosine, even in the background of relatively low total ND, can have a significant impact on protein structure and function.

**Figure 1:**
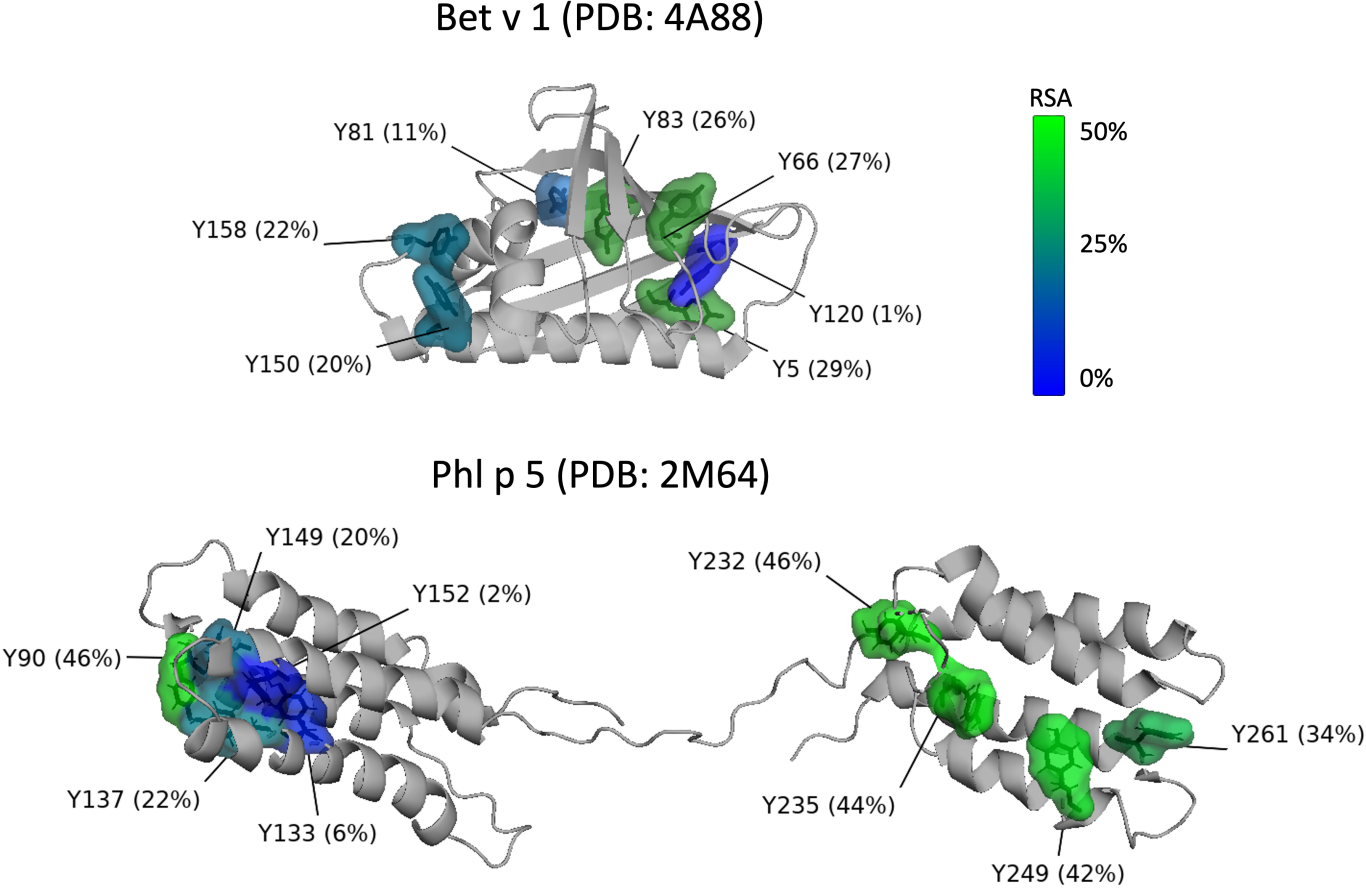
Position and relative solvent accessibility (RSA) of the tyrosine residues in the 3D-structure (ribbon view) of Bet v 1.0101 (PDB accession code: 4A88) (56) and Phl p 5.0101 (PDB accession code: 2M64, assembly 1) (57) created with the PyMOL Molecular Graphics System, Version 2.5.2 Schrödinger, LLC (58). Tyrosine residues are displayed as molecular surface and numbered according to the amino acid sequence of full-length Bet v 1 and Phl p 5. Coloring of the tyrosine residues is according to their RSA from blue (0%, buried) to green (50%, exposed).

Figure 1 shows the positions and the relative solvent accessibility (RSA) of the tyrosine residues for both allergens based on protein databank entries PDB 4A88 (Bet v 1) and PDB 2M64 (Phl p 5) (56, 57). Bet v 1 consists of 159 amino acids and contains seven tyrosine residues (Y5, Y66, Y81, Y83, Y120, Y150, Y158) as potential nitration sites. Based on the RSA values, all tyrosine residues from Bet v 1 are well accessible for modification, with the exception of Y120. This is in agreement with Karle et al. (9), who predicted for another databank entry of Bet v 1 (PDB 1BV1) and based on accessibility and electrostatics that Y5, Y81, Y83, Y150, and Y158 are preferentially nitrated, while the positively charged environment of Y66 disfavors nitration, and Y120 is inaccessible due to its shielded position. Gusenkov et al. (59) characterized ONOO${}^{-}$-modified Bet v 1 (at ONOO${}^{-}$ to tyrosine molar ratios of 1:1 and 5:1) and could distinguish up to 12 variants with one to sixfold nitration. The three- to sixfold nitrated variants were detected only in samples modified with higher amounts of ONOO${}^{-}$ (molar ratio 5:1). Their results indicate the occurrence of Bet v 1 variants with identical nitration degrees but site-specific nitration.

Reinmuth-Selzle et al. (29) found that the preferred reaction sites of Bet v 1 vary depending on the nitrating agent. Residues Y81 and Y83 were the preferred nitration sites for TNM, Y83 and Y158 for O${}_{3}$/NO${}_{2}$, and Y150 for ONOO${}^{-}$. These tyrosine residues have high solvent accessibility and are located in hydrophobic protein environments. Residues Y150 and Y158 are located in the C-terminal helix and Y81 and Y83 in the hydrophobic cavity, both key positions for the binding of specific IgE as well as ligands such as fatty acids, cytokines, and flavonoids (29). Low levels of nitration were achieved for Y81 (O${}_{3}$/NO${}_{2}$), Y150 (TNM, O${}_{3}$/NO${}_{2}$), and Y158 (ONOO${}^{-}$). Y5 and Y120 nitration could not be detected and Y66 nitration was only found for ONOO${}^{-}$-modified Bet v 1, but could not be quantified. Gusenkov and Stutz (60) later found that the reaction with ONOO${}^{-}$ leads to nitration of six tyrosine residues (Y5, Y66, Y81, Y83, Y150, Y158) with preferential nitration of the two surface-exposed tyrosine residues Y5 and Y66.

Site-specific nitration and different variants can also be expected for Phl p 5, but so far, to our knowledge, the preferred nitration sites have not been determined for the grass pollen allergen. After cleavage of the 25 amino acid signal peptide, the mature Phl p 5 consists of 287 amino acids including 12 tyrosine residues: Y30, Y42, Y90, Y133, Y137, Y149, Y152, Y232, Y235, Y249, Y261, and Y310. The 3-D ribbon structure of Phl p 5 is shown in Figure 1. Because the available structure covers only the amino acid residues 55 to 285 only nine tyrosine residues (Y90-Y261) are included. The protein consists of two domains that are flexibly connected by a central linker region. Each domain is formed by a 4-helix bundle stabilized by a hydrophobic core (57).

Applying the commonly used RSA threshold of 20% to define buried and exposed residues, two out of the nine tyrosine residues (Y133, Y152) can be classified as buried, while the other seven residues (Y90, Y137, Y149, Y232, Y235, Y249, Y261) are surface exposed and can be expected to be more susceptible to nitration (55, 61, 62). Further studies are needed to determine the preferred nitration sites in the grass pollen allergen.

## 3. Dityrosine cross-linking

Dityrosine cross-linking can lead to protein oligomerization and the formation of insoluble protein. In contrast to tyrosine nitration, less is known about the oligomerization via dityrosine cross-linking for the two allergens as the initial studies focused on tyrosine nitration. However, dimer and trimer formation was observed but not quantified for the reaction of Bet v 1 with TNM (9, 35). The reaction of Bet v 1 with ONOO${}^{-}$ yielded up to 42% protein dimers and higher oligomers (63). For Bet v 1, the tyrosine residues, Y5, Y66 and Y150 were suggested to be involved in the dityrosine cross-linking due to their solvent-exposed position (35, 64).

For Phl p 5, exposure to O${}_{3}$/NO${}_{2}$ and ONOO${}^{-}$ lead to the formation of protein dimers and higher oligomers for up to 50% of the protein mass (30, 63). For dimerization and further oligomerization, the effect of steric hindrance, however, is likely more important than for the nitration reaction (22). Thus, for Phl p 5, the tyrosine residues Y90, Y232, Y235, Y249, and Y261 could potentially be involved in dityrosine formation due to their high solvent exposure (Figure 1). For both allergens, further studies are needed to determine the preferred reaction sites for dityrosine cross-linking and to investigate the extent of cross-linking under different reaction conditions. Since different structural dimers may have different biological effects, a site-selective characterization is also required for dityrosine cross-links, similar to the concept of the tyrosine-specific degree of nitration.

## 4. Influence of the reaction conditions on tyrosine nitration and dityrosine formation

Several studies have investigated the influence of experimental conditions on the extent of tyrosine nitration and dityrosine cross-linking. For example, for the reaction with TNM, the ND of Bet v 1 strongly depends on the molar ratio of TNM to tyrosine residues (29), whereas the reaction time seems to be less important (51). Similarly, also for the reaction with ONOO${}^{-}$, the ND depends on the molar ratio of ONOO${}^{-}$ to tyrosine residues (29, 30, 63). In contrast to the reaction with TNM, the ND for the reaction of Bet v 1 with ONOO${}^{-}$ depends on the reaction time. Shorter reaction times (15 min vs. 100 min) lead to higher NDs and, in addition, low temperatures (4${}^{\circ }$C vs. RT) and the addition of a chelator diethylenetriamine pentaacetic acid (DTPA), which prevents the reaction of ONOO${}^{-}$ with metal ions, such as iron or copper, favor nitration (29). Reaction time and temperature affect the generally rapid degradation of ONOO${}^{-}$ and the degree of protein degradation, which increases with increasing reaction time and temperature.

The reaction with O${}_{3}$/NO${}_{2}$ is strongly influenced by changes in the O${}_{3}$ concentration and rather insensitive to changes in the NO${}_{2}$ concentration (30, 65). For the grass pollen allergen Phl p 5, higher nitration and oligomerization was observed at higher gas concentrations and longer reaction times, but the highest oligomerization was achieved for the reaction with O${}_{3}$ alone (30). This can be explained by a competitive reaction of the tyrosyl radical formed in a first reaction step with O${}_{3}$, which either reacts with an NO${}_{2}$ molecule, leading to the formation of 3-nitrotyrosine, or with another tyrosyl radical, forming a dityrosine cross-link (22, 66). Without the addition of NO${}_{2}$, there is no competitive reaction resulting in higher oligomerization of the protein. Higher temperatures can increase the rate of tyrosyl radical formation by ozonolysis, contributing to the formation of nitrated and cross-linked protein species, especially under tropical and summer smog conditions (21, 65, 67). In this context, also the lifetime of an allergen in the air becomes important as a longer atmospheric residence time increases the chances for chemical modification by O${}_{3}$ and NO${}_{2}$.

Under atmospheric conditions, the reaction rates also depend on the phase state of the proteins which, in turn, depends on temperature and relative humidity. At high temperature and high humidity, the phase state of atmospheric particles is liquid, changing to viscous, semi-solid, or even glassy at low temperature and low humidity (66, 68, 69). In the liquid state, O${}_{3}$ and NO${}_{2}$ diffuse faster into proteins and can therefore react faster than with solid or semi-solid proteins (70). For reactions of Bet v 1 with atmospherically relevant concentrations of O${}_{3}$/NO${}_{2}$ in the aqueous phase, the nitration rate was one order of magnitude higher (ND ≈ 20% per day) than for solid and semi-solid proteins on filter samples (ND ≈ 2% per day), indicating an increased relevance of these processes under humid summer smog conditions (29). For solid or semi-solid proteins, such as protein films on the surface of aerosol particles at low relative humidity, the tyrosine residues near the surface are expected to be nitrated more efficiently since the nitration of tyrosine residues in the bulk of the protein film is kinetically limited by the diffusivity of O${}_{3}$ and NO${}_{2}$ (22, 71).

## 5. Nitration and dityrosine cross-linking increase allergenicity of Bet v 1 and Phl p 5

For TNM-modified Bet v 1, several effects on the immune response have been reported. These include increased proliferation of Bet v 1-specific T cells, greater proteolytic resistance, enhanced antigen presentation, altered cytokine profiles, increased IgE binding, and mediator release (8, 9, 35). Bet v 1 is a small protein with several IgE binding sites clustered in a small area (64, 72–75). Protein dimers and oligomers may play an important role in the reported changes in allergenicity because they display repetitive epitopes. Repetitive epitope presentation is required for the cross-linking of B-cell receptors and activation of B-cells and IgE production, and also facilitates cross-linking of the effector cell-bound IgE, a key event in the initiation of the allergic response (76–80). Aggregation and adsorption to natural or anthropogenic particles can also lead to the display of multiple epitopes (10). In addition to repetitive epitope presentation, nitration and dityrosine cross-linking can lead to epitope modification or shielding, or neoepitope formation (8, 10, 78, 81, 82).

**Figure 2:**
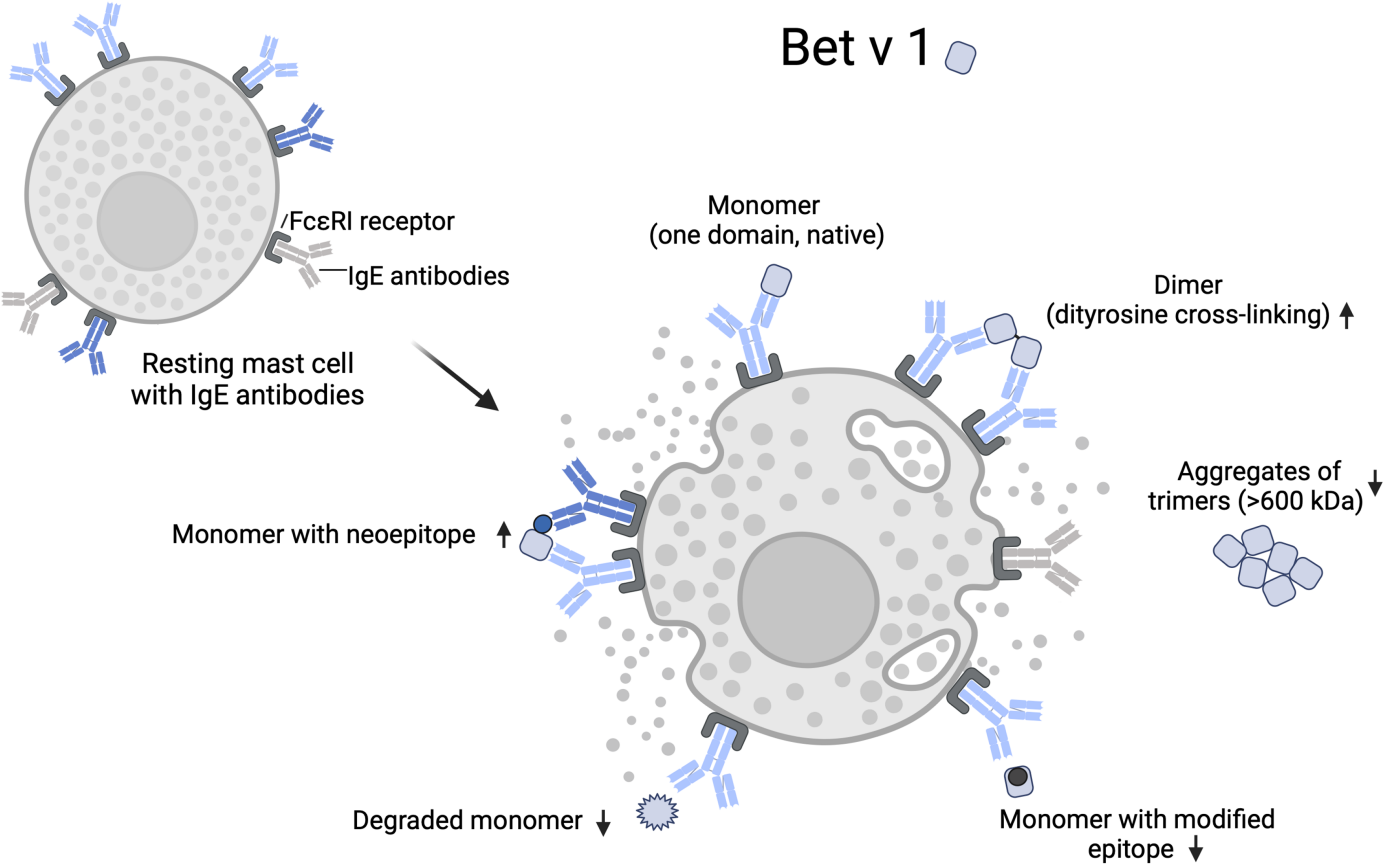
Mast cell degranulation by native and modified Bet v 1. IgE cross-linking on effector cells requires binding to at least two epitopes. Chemical modification can lead to an increase or decrease of accessible epitopes on an allergen through nitration, intra- or intermolecular dityrosine cross-linking, conformational changes, and protein degradation. Arrows indicate a relative increase or decrease of IgE cross-linking by individual allergen variants. Modification of epitope sites and shielding of epitopes by aggregation reduces IgE cross-linking, while the formation of neoepitopes can increase the IgE cross-linking if specific antibodies are bound to the mast cell. Created with BioRender.com.

Figure 2 illustrates how the native (monomeric) and modified Bet v 1 variants may increase or decrease cross-linking of mast cell-bound IgE. Binding of monomeric Bet v 1 does not induce cross-linking of IgE binding to the same epitope. Bet v 1 dimers formed by chemical modification can provide two epitope sites that facilitate IgE cross-linking. Binding of monomers may also lead to mast cell degranulation at high allergen concentrations if two monomers bind in close proximity to each other and dimerize on the mast cell surface (76). Bet v 1 monomers could also cross-link IgE if the mast cell carries IgEs, whose binding sites on the monomer do not overlap. This would be more likely if structural changes or nitration lead to the formation of neoepitopes, such as haptenic nitroaromatic groups, which are particularly small and highly immunogenic (83).

In sera of allergic patients, Gruijthuijsen et al. (8) found higher levels of IgE specific for the TNM-modified Bet v 1 than for the native Bet v 1. This suggests that some patients may have been exposed and sensitized to different variants of Bet v 1, as their sera contained IgE specific for the neoepitopes of Bet v 1. Chemical modification of epitope sites can theoretically also lead to reduced IgE cross-linking if the IgE are specific for the unmodified epitope or if oligomerization leads to altered epitope presentation or shielding by steric hindrance and/or altered charge interactions (84, 85). For example, reduced basophil activation and mediator release has been reported for trimeric Bet v 1, which tends to form high molecular weight aggregates in solution likely reducing the number of displayed epitopes (86).

Recognition of allergens by airway epithelial receptors such as the Toll-like receptor 4 (TLR4) and other direct allergen-airway epithelial interactions are the first events following allergen inhalation. TLR4 activation has been found for the grass pollen allergen Phl p 5 but not for the birch pollen allergen Bet v 1 (63). TLR4 activation leads to the release of cytokines and other danger signals that initiate the presentation of the allergen to immune cells and the production of allergen-specific IgE, which is crucial for the allergic response phase (63, 87–92). Peroxynitrite modification enhanced TLR4 activation of Phl p 5 by factors of up to ∼ 2.1 (63). This may promote sensitization to the grass pollen allergen under conditions of oxidative stress through positive feedback loops (31, 63, 93).

## 6. Future research

Understanding how environmental risk factors such as air pollution affect the allergenic potential of proteins, either directly or indirectly via oxidative stress, is crucial for the protection of public health (10, 69, 94). The existing literature shows that the reaction of Bet v 1 and Phl p 5 with O${}_{3}$/NO${}_{2}$ or ONOO${}^{-}$ can result in various mixtures of nitrated and cross-linked variants with altered allergenicity. Potential real-world exposure and variations in the allergenic potential among the nitrated and cross-linked variants remain unclear. Future studies should investigate the extent of allergen modification, both within pollen and as free allergens under environmental conditions. Because allergen content and release from pollen vary, airborne allergens and their variants should be monitored (95–98). In vitro and in vivo studies are needed to assess the allergenic potential of the individual variants and their mixtures in the development and response phases of allergic diseases. In addition, research is needed to investigate the importance of photo-oxidation and the reaction of allergenic proteins with the air pollutant peroxyacetyl nitrate on tyrosine nitration and dityrosine cross-linking (23, 24, 27).
